# Romantic relationships and adolescent depression in China: moderating effects of peer norms

**DOI:** 10.3389/fpsyt.2025.1644371

**Published:** 2025-07-30

**Authors:** Anqin Zhu, Zeyu Kong, Aiping Zhou

**Affiliations:** ^1^ School of Government, Nanjing University, Nanjing, China; ^2^ Center for Health Policy and Management Studies, School of Government, Nanjing University, Nanjing, China; ^3^ Nanjing Drum Tower Hospital, The Affiliated Hospital of Nanjing University Medical School, Nanjing, China

**Keywords:** adolescent depression, mental health, romantic relationships, peer norms, descriptive norms, injunctive norms

## Abstract

**Objective:**

Adolescent depression is a crucial determinant of an individual’s developmental trajectories and long-term health-related quality of life. Romantic relationships are widely recognized as a risk factor of adolescent depression. Peer norms, as an important source of social influence in adolescence, have the potential to moderate the effects of romantic relationships. The present study aims to examine the moderating effects of peer norms, including descriptive norms and injunctive norms, on the association between romantic relationships and adolescent depression in China.

**Methods:**

The present study used a nationally representative sample of 6718 junior high school students from the China Education Panel Survey (CEPS). The ordinary least squares regression model was used to assess both the main effects of romantic relationships on adolescent depression and the moderating effects of peer norms. The bounding approach was used for sensitivity analysis.

**Results:**

Romantic relationships were positively associated with adolescent depression (β=0.290, p<.001). Descriptive norms, including class norms (β=-0.634, p=.042) and friend norms (β=-0.206, p<.001), significantly mitigate the association between romantic relationships and adolescent depression, but injunctive norms (β=0.253, p=.035) significantly exacerbate the association. The sensitivity analysis reveals that the above results are robust to the potential confounding impact of unobserved variables.

**Conclusion:**

Peer norms were found to significantly moderate the correlation between romantic relationships and adolescent depression. It is important to play the role of descriptive and injunctive peer norms in the prevention and intervention for the depression of adolescents, especially for those who are in a romantic relationship.

## 1 Introduction

Depression in adolescence, as a crucial determinant of an individual’s developmental trajectories and long-term health-related quality of life, is a serious public health challenge globally with a worldwide prevalence of 6% (1–3). Due to the lack of mental health education and the stigma attached to mental illness, the prevalence of depression among Chinese adolescents is about 24.3%, which is much higher than the global level (4). Depression can impair adolescents’ physical and mental health, social adjustment, and interpersonal interactions, leading to poor academic performance, peer ostracism, and even suicidal behavior (5–10). Additionally, the negative effects of adolescent depression can persist over time, increase the risk of depression in adulthood, and have significant economic and social consequences (11). Therefore, it is crucial to prevent and intervene in this disorder.

Adolescence is a period during which romantic relationships emerge, and most teenagers will experience their first love in adolescence (12). Several studies have found that romantic relationships were positive impact on adolescents (13), who may experience emotional support from their partners and an increased sense of self-worth during romance (14). However, since Joyner and Udry’s influential work (15), researchers have noted the negative effects of romantic relationships on mental health of adolescents. Emerging studies suggest a link between adolescent romantic experiences and depression (16–18). Specifically, romantic relationships divert adolescents’ attention from other important developmental tasks (e.g., academics, family relationships, and friendships), thereby undermining adjustment in these areas and increasing risk for depression (15, 19). Furthermore, previous studies showed that depressive disorders may result from responding to current circumstances and stress (20). Romantic relationships will bring new types of stressors to adolescents, including communication with heterosexual partners, exposure to interpersonal conflict, and suffering from physical or relational aggression, etc. (21). Adolescents in romantic relationships who lack the coping abilities and resources necessary to manage these stresses will be at higher risk for depression (22–26). Additionally, adolescents involved in non-normative and off-time romantic activities would be more likely to be maladjusted and depressed due to their unpreparedness for advanced relationships and lack of peer support (23).

As adolescent depression is becoming more prevalent and causes a serious personal and societal burden (27), there is a need to explore the moderators of the link between romantic relationships and adolescent depression. According to the social ecological theory, depression is the product of complex interaction of individual and environmental characteristics (28). Among the potentially influential environmental features are family dynamics, peer groups, and school contexts. During adolescence, individuals spend more time with peers and are more inclined to conform to the views of peers than parents and teachers (29). In other words, the link between romantic relationships and adolescent depression may be conditioned by peer norms (10, 23). From the perspective of social norm theory, there are two types of peer norms: descriptive norms, which are defined as actual or perceived behaviors of peers, and injunctive norms, which are defined as actual or perceived attitudes of peers regarding the engagement in specific behaviors (30–32). Recent studies have explored the moderating role of descriptive peer norms in the relationship between adolescent romantic involvement and depression (9, 10), but limitations remain. First, while these studies have investigated the impact of romantic experiences on adolescents’ depressive symptoms depended on two types of descriptive norms, including class norms and friend norms (9, 10), injunctive norms have not been taken into consideration. Second, although friend networks of teenagers often extend far beyond the classroom, friend norms have been confined to the in-class groups in previous studies (10). Third, existing studies have failed to provide causal evidence due to the potential confounding impact of unobserved variables (e.g., genetic and environmental factors), and may thus inadequately account for the influence of peer norms on association between romantic relationships and adolescent depression.

In the present study, we used data from a large, nationally representative sample of adolescents in China. to investigate how peer norms moderate the relationship between romantic relationships and adolescent depression. We examined whether the proposed moderating effects of descriptive norms on the link between romantic relationships and depression applies to two types of peer networks, including class networks and friendship networks. And we further tested the moderating effects of injunctive norms, thereby providing a complete picture of the role of peer norms. Additionally, we used the bounding approach to rule out potential endogeneity due to omitted variable bias. Such efforts will inform provocative depression interventions for adolescents in a romantic relationship.

## 2 Methods

### 2.1 Data

**Figure 1:**
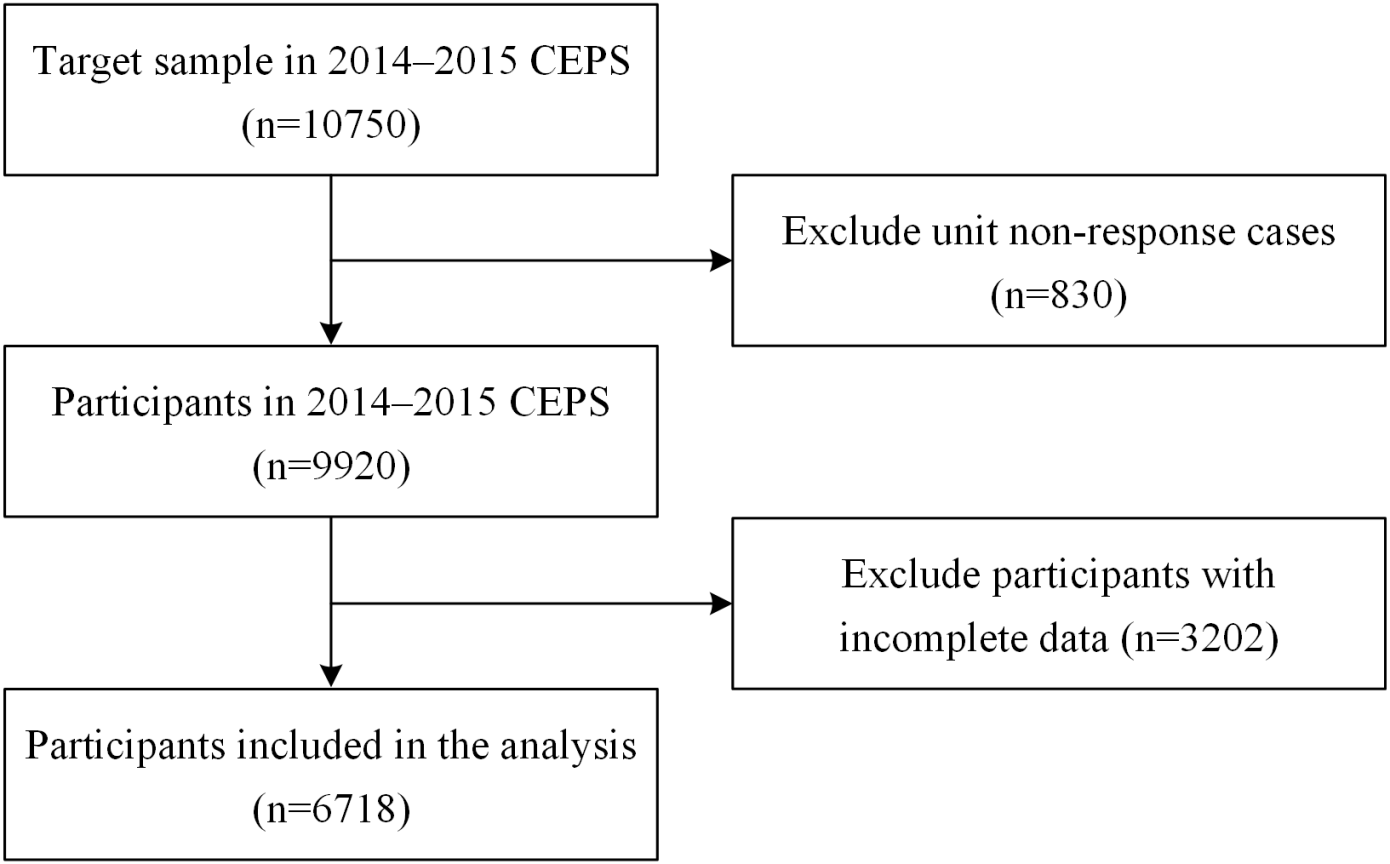
Flowchart of sample selection and exclusion.

The China Education Panel Survey (CEPS) is a nationally representative social survey implemented by the National Survey Research Center (NSRC) of Renmin University of China. It investigated the junior high school students in 28 counties, 112 schools, and 438 classes across the country. Research ethics approval for data collection of CEPS study was granted by the Institutional Review Board of Renmin University of China, and the informed consent was required to sign by the participants. The wave in 2014–2015 school year is the latest wave of the data and includes a target sample of 10750 individuals. The information collected included the characteristics of students, parents, classes, and schools. Due to student absences caused by leave requests, school transfers, and dropouts, the unit non-response rate in the 2014–2015 CEPS dataset was relatively high (approximately 7.7%). Additionally, since this study employs the bounding approach to test the sensitivity of regression results to omitted variable bias, it is necessary to control potential confounding variables as much as possible, resulting in a relatively small complete-case sample. However, using imputation methods to handle missing data may introduce estimation bias due to non-random missingness. Accordingly, following established practices in the literature (9, 10, 33), this study applied listwise deletion to handle missing values. Specifically, samples with missing values in the dependent variable, independent variables, moderating variables, or control variables were excluded, yielding a final complete-case sample of 6718 observations for subsequent empirical analysis. The flowchart of sample selection and exclusion is presented in **Figure 1**. Additionally, to address concerns about sample loss due to listwise deletion, a *post-hoc* power analysis was conducted, showing that at the 0.05 significance level, the statistical power of both the main effects model and the moderating effects model exceeded 99%. This indicates that even with listwise deletion, the regression models maintain high statistical power, thus supporting the reliability of the estimation results.

### 2.2 Variables

#### 2.2.1 Dependent variable

Adolescent depression was measured by the 10-item scale. Respondents were asked how often they had the following emotions on a 5-points scale (1=never, 5=always): “dispirited”, “downhearted”, “unhappy”, “meaningless”, “exhausted”, “sad”, “anxious”, “worried”, “ominous”, and “inattentive”. Cronbach’s alpha was 0.91. Adolescent depression was measured with a mean of these 10 items.

#### 2.2.2 Independent variable

Romantic relationship was evaluated by the item “have you ever engaged in a romantic relationship?”. Romantic relationships were coded as 1 if the respondents answered “yes”, and 0 if they answered “no”.

#### 2.2.3 Moderating variables

Peer norms included descriptive norms and injunctive norms. Descriptive norms were distinguished into class norms and friend norms. The former was measured with the proportion of adolescents in the same class who ever engaged in a romantic relationship. The latter was measured by the item “how many of your five best friends have the following behavior? To engage in a romantic relationship” represented on a 3-points scale (0=none, 1=one or two, 2=a lot). Regarding injunctive norms, based on data availability and following existing approaches in the literature (34–36), this study measured them using respondents’ perceptions of their peers’ attitudes toward adolescent romance. These perceptions were coded as 1 if the respondent indicated “objection” and 0 otherwise. The rationale for employing a binary variable is that it effectively captures the qualitative differences inherent in injunctive norms. Methodological studies have demonstrated that using a binary measure does not substantially reduce statistical power, while also simplifying the analysis and interpretation of interaction effects. This approach allows for a clearer distinction between different types of interaction effects (37). Therefore, measuring injunctive norms with a single binary item is highly justifiable and methodologically sound.

#### 2.2.4 Control variables

We controlled for the following variables: gender (female, male), age, ethnicity (minority, Han), hukou type (agricultural hukou, non-agricultural hukou, unified residence hukou), migration status (migrant, local), family structure (single-parent family, two-parent family), number of siblings, parental education (higher value of the years of education between parents), family socio-economic status (1=very poor, 5=very rich), number of books (1=very few, 5=a lot), study desk (no, yes), number of friends, gender of homeroom teacher (female, male), age of homeroom teacher, education of homeroom teacher (in years), teaching experiences of homeroom teacher (in years), class rank (1=worst, 5=best), class size (number of students of class), school type (private, public), school rank (1=worst, 5=best), school size (number of students of school). Additionally, county fixed effects were controlled to rule out the influence of unobservable regional characteristics.

### 2.3 Statistical analysis

The characteristics of students were described by means and standard deviations (SD) for numeric variables and numbers and percentages for categorical variables. The differences in characteristics between adolescents in a romantic relationship and those who were not were evaluated using t-tests and Chi-square tests. Ordinary least squares (OLS) regression model was used to estimate the main effects of romantic relationships on adolescent depression and the moderating effects of peer norms. In case there may be unobservable variables that can affect both romantic relationships and adolescent depression, the bounding approach was used to test the stability of coefficients and confirm the robustness of baseline regression results. Stata SE 17 software (Stata Corp LP, College Station, TX, USA) was used to conduct all analyses and p < .05 was considered as statistical significance.

## 3 Results

### 3.1 Sample characteristics

The descriptive statistics in **Table 1** show that a total of 6718 adolescents were included, with a mean age of 13.89 (SD 0.83) years and a female proportion of 49.69%. There were 785 adolescents who had been in a romantic relationship and 5933 who had not. On average, adolescents who had been in a romantic relationship had significantly higher depression scores than those who had not (p<.001). Compared to adolescents who had not been in a romantic relationship, adolescents who had been in a romantic relationship were more likely to be in peer networks where romantic relationships are more common and less objectionable, as evidenced by more positive class norms (p<.001), friend norms (p<.001) and injunctive norms (p<.001). Additionally, adolescents who had been in a romantic relationship were more likely to be male (p<.001), older (p<.001), migrant (p=.004), live in single-parent family (p=.001), have more siblings (p<.001), not have much books (p=.002) or study desk (p=.045) at home, and have more friends (p=.011). Moreover, from the perspective of class and school situation, for teenagers with romantic experiences, their homeroom teachers are more likely to be male (p<.001), and their schools are more likely to be private (p<.001), and lower-ranked (p<.001).

**Table 1:** Descriptive statistics.

Variables	Total (n=6718)		Romantic relationship group (n=785)		Non-romantic relationship group (n=5933)		p
	Mean/n	SD/%	Mean/n	SD/%	Mean/n	SD/%	
Panel A. Numeric variables							
Depression	2.16	0.80	2.43	0.83	2.13	0.79	<.001
Class norms	0.12	0.09	0.16	0.10	0.12	0.09	<.001
Friend norms	0.33	0.54	0.90	0.64	0.25	0.48	<.001
Age	13.89	0.83	14.11	0.85	13.87	0.83	<.001
Number of siblings	0.69	0.77	0.80	0.85	0.67	0.75	<.001
Parental education	11.09	3.05	10.91	3.05	11.11	3.04	.073
Family socio-economic status	2.96	0.59	2.98	0.65	2.96	0.58	.246
Number of books	3.08	1.15	2.96	1.21	3.10	1.14	.002
Number of friends	9.14	10.56	10.04	10.98	9.02	10.50	.011
Age of homeroom teacher	38.59	6.78	38.59	7.08	38.60	6.74	.986
Education of homeroom teacher	15.94	0.55	15.90	0.68	15.94	0.53	.059
Teaching experiences of homeroom teacher	16.53	8.13	16.20	8.24	16.57	8.11	.224
Class rank	3.43	1.00	3.41	0.98	3.44	1.00	.475
Class size	45.47	14.97	46.06	15.28	45.39	14.93	.240
School rank	3.98	0.86	3.84	0.95	4.00	0.85	<.001
School size	1128.71	692.18	1086.41	700.25	1134.30	690.97	.069
Panel B. Categorical variables							
Injunctive norms							<.001
No objection	5573	82.96	741	94.39	4832	81.44	
Objection	1145	17.04	44	5.61	1101	18.56	
Gender							<.001
Female	3338	49.69	322	41.02	3016	50.83	
Male	3380	50.31	463	58.98	2917	49.17	
Ethnicity							.081
Minority	434	6.46	62	7.90	372	6.27	
Han	6284	93.54	723	92.10	5561	93.73	
Hukou type							.348
Agricultural hukou	3506	52.19	427	54.39	3079	51.90	
Non-agricultural hukou	1844	27.45	200	25.48	1644	27.71	
Unified residence hukou	1368	20.36	158	20.13	1210	20.39	
Migration status							.004
Migrant	1148	17.09	163	20.76	985	16.60	
Local	5570	82.91	622	79.24	4948	83.40	
Family structure							.001
Single-parent family	559	8.32	89	11.34	470	7.92	
Two-parent family	6159	91.68	696	88.66	5463	92.08	
Study desk							.045
No	1375	20.47	182	23.18	1193	20.11	
Yes	5343	79.53	603	76.82	4740	79.89	
Gender of homeroom teacher							<.001
Female	4365	64.97	460	58.60	3905	65.82	
Male	2353	35.03	325	41.40	2028	34.18	
School type							<.001
Private	412	6.13	87	11.08	325	5.48	
Public	6306	93.87	698	88.92	5608	94.52	

### 3.2 Regression analysis of the main effects of romantic relationships on adolescent depression and the moderating effects of peer norms

The main effect model in **Table 2** showed that romantic relationships were significantly positively associated with adolescent depression (β=0.290, p<.001), which suggests that adolescents who engaged in a romantic relationship experienced more depressive affects. Additionally, several control variables, which include being male (β=-0.043, p=.030), living in a two-parent family (β=-0.149, p<.001), higher family socio-economic status (β=-0.090, p<.001), more books (β=-0.053, p<.001), more friends (β=-0.004, p<.001), higher class rank (β=-0.023, p=.031) and public school (β=-0.140, p=.007) were found to significantly decrease adolescents depression, while being older (β=0.032, p=.037), unified residence hukou (β=0.060, p=.040), a male homeroom teacher (β=0.045, p=.047) and higher school rank (β=0.047, p=.006) were found to significantly increase adolescents depression. The effects of other control variables were not statistically significant.

**Table 2:** Regression results of the main effects of romantic relationships on adolescent depression and the moderating effects of peer norms.

Variables	Main effect model		Moderating effect model	
	β	SE	β	SE
Romantic relationships	0.290^***^	0.031	0.404^***^	0.076
Romantic relationships × class norms			-0.634^*^	0.312
Romantic relationships × friend norms			-0.206^***^	0.053
Romantic relationships × injunctive norms			0.253^*^	0.120
Class norms			-0.041	0.138
Friend norms			0.249^***^	0.025
Injunctive norms			-0.056^*^	0.026
Gender	-0.043^*^	0.020	-0.057^**^	0.020
Age	0.032^*^	0.015	0.027	0.015
Ethnicity	-0.054	0.047	-0.057	0.046
Hukou type (ref: agricultural hukou)				
Non-agricultural hukou	-0.009	0.029	-0.015	0.029
Unified residence hukou	0.060^*^	0.029	0.058^*^	0.029
Migration status	0.014	0.028	0.015	0.028
Family structure	-0.149^***^	0.037	-0.141^***^	0.037
Number of siblings	0.030	0.016	0.026	0.015
Parental education	0.001	0.004	0.001	0.004
Family socio-economic status	-0.090^***^	0.021	-0.090^***^	0.021
Number of books	-0.053^***^	0.011	-0.051^***^	0.011
Study desk	-0.036	0.029	-0.029	0.029
Number of friends	-0.004^***^	0.001	-0.004^***^	0.001
Gender of homeroom teacher	0.045^*^	0.023	0.039	0.023
Age of homeroom teacher	-0.003	0.004	-0.003	0.003
Education of homeroom teacher	0.014	0.021	0.018	0.021
Teaching experiences of homeroom teacher	0.001	0.003	0.002	0.003
Class rank	-0.023^*^	0.010	-0.024^*^	0.010
Class size	0.001	0.001	0.001	0.001
School type	-0.140^**^	0.052	-0.159^**^	0.054
School rank	0.047^**^	0.017	0.041^*^	0.017
School size	-0.000	0.000	-0.000	0.000
Intercept	2.156^***^	0.443	2.151^***^	0.439
Observations	6718		6718	
R^2^	0.064		0.086	

^*^ *p* < 0.05, ^**^ *p* < 0.01, ^***^ *p* < 0.001.

The moderating effect model in **Table 2** showed that the relationship between romantic relationships and adolescent depression was moderated by peer norms. Specifically, descriptive norms, including class norms (β=-0.634, p=.042) and friend norms (β=-0.206, p<.001), acted as negative moderators, whereas injunctive norms (β=0.253, p=.035) showed positive moderating effects. In other words, the effect of romantic relationships on adolescent depression will decrease as romantic involvement become more common in peer networks and intensify when peers oppose adolescent romance.

Following the approach of Guriev et al. (38) and Mitton (39), this study further interprets the clinical effect sizes of both the main effect of romantic relationships on adolescent depression and the moderating effects of peer norms. Regarding the main effect, the analysis indicates that involvement in a romantic relationship increases the level of adolescent depression by approximately 13.6% (0.290 ÷ 2.13). For comparison, the coefficient for family structure in the same regression is -0.149, suggesting that the impact of romantic involvement on adolescent depression is nearly twice as large as the effect of living in a single-parent family. Regarding the moderating effect, a one standard deviation increase in classroom norms reduces the effect of romantic relationships on depression by 19.7% (−0.634 × 0.09 ÷ 0.290); a one-unit increase in friend norms reduces this effect by 71.0% (−0.206 ÷ 0.290); and injunctive norms—namely, peers’ objection to adolescent romantic relationships—amplify this effect by 87.2% (0.253 ÷ 0.290). Taken together, the magnitudes of both the main and moderating effects are substantial and merit careful consideration in both research and practice.

### 3.3 Sensitivity analysis

The baseline regression results may be confounded by unobservable variables, such as genetic and environmental factors, potentially leading to spurious correlations. To address the endogeneity due to omitted variables bias, we conducted a sensitivity analysis using the bounding approach of Oster (40) to examine the robustness of the baseline results.

The bounding approach requires two key pieces of information. The first is the selection ratio δ, which refers to the explanatory power of the observable variables relative to the unobservable variables. The second is the maximum R^2^ (denoted by R_max_^2^) obtained from a hypothetical regression controlling for both observable and unobservable variables. Following the suggestions of existing studies (40), we took two approaches to conduct sensitivity analysis. First, given the values of R_max_^2^ and δ, we could obtain the identified set (or bounded set) defined by (β, β* (Min {1, 1.3R^2^}, δ=1)) which contains true estimates. If the identified set does not include zero, then the estimation results of the baseline regressions are robust to omitted variable bias. Second, given the value of R_max_^2^, the value of δ when the treatment effect is 0 could be obtained. If |δ|> 1, the results are robust.

The results of the sensitivity analysis in **Table 3** showed that the identified sets for both the main effects of romantic relationships on adolescent depression and the moderating effects of class norms, friend norms and injunctive norms did not include zero, and all the absolute values of selection ratios (|δ|) were greater than 1, implying that the baseline results were robust to endogeneity in terms of omitted variable bias.

**Table 3:** Sensitivity analysis.

Variables	Controlled effect		Identified set	δ
	β	SE		
Panel A. Main effect model				
Romantic relationships	0.290^***^	0.031	[0.283, 0.290]	16.372
Observations	6718			
R^2^	0.064			
Panel B. Moderating effect model				
Romantic relationships	0.404^***^	0.076	[0.388, 0.404]	17.199
Romantic relationships ×class norms	-0.634^*^	0.312	[-0.634, -0.613]	21.266
Romantic relationships ×friend norms	-0.206^***^	0.053	[-0.206, -0.192]	12.575
Romantic relationships ×injunctive norms	0.253^*^	0.120	[0.253, 0.259]	-50.150
Observations	6718			
R^2^	0.086			

^*^ *p* < 0.05, ^**^ *p* < 0.01, ^***^ *p* < 0.001.

## 4 Discussion

### 4.1 Main effects of romantic relationships on adolescent depression

Adolescence is a time when depression rates increase. Depression is likely to affect adolescents’ academic performance, interpersonal functioning, and subjective well-being. Romantic relationships are found to be one of the most important predictors of depression in adolescence. Using nationally representative samples in China, the current study explored whether the association between romantic relationships and adolescent depression was conditioned by peer norms. Results suggested that both descriptive and injunctive peer norms played the moderating roles in the relationship between romantic relationships and adolescent depression. In addition, the above conclusions still hold after using bounding approach to rule out potential endogeneity due to omitted variable bias.

Theoretically, the association between romantic relationships and adolescent depression remain inconclusive. Most prevailing theoretical models suggest that romantic relationships may exacerbate adolescent depression. For instance, according to the normative trajectory model, adolescents who engage in non-normative activities tend to exhibit poorer socio-emotional health compared to those who follow normative developmental pathways. As romantic involvement during adolescence is commonly perceived as non-normative and off-time behavior, adolescents who participate in such relationships are more likely to experience heightened depression (6, 9). Similarly, the attention impairment model posits that romantic relationships, as an emerging developmental task, divert adolescents’ attention from other important domains such as academics, family, and friendships. This diversion can undermine their adjustment in these areas and increase the risk of depression (15, 19). Furthermore, the stress and coping model suggests that romantic relationships introduce novel stressors—such as interpersonal conflicts and breakups—which elevate overall stress levels and may contribute to greater negative psychological impact among adolescents who lack the coping skills and resources necessary to manage these challenges (25). However, alternative theoretical perspectives emphasize the positive role of adolescent romantic relationships in psychosocial adjustment. From the perspective of the bottom-up theory of happiness, the accumulation of positive emotional experiences may foster an optimistic outlook and a sanguine temperament (41). Within romantic relationships, adolescents may receive emotional support from their partners and experience positive emotional feedback, which could buffer against negative emotions, thereby promoting psychological well-being (13, 14).

Currently, empirical studies conducted in Western countries have documented a significant positive association between romantic relationships and adolescent depression (15–18). The present study contributes to this body of literature by providing cross-national evidence supporting the negative psychological impact of adolescent romantic relationships. However, it is important to note that due to data limitations, the current study was unable to account for adolescents’ emotional experiences during romantic relationships—such as joy, satisfaction, and fulfillment—and thus cannot fully rule out the existence of such mechanisms. Consequently, our findings be interpreted as a reduced-form estimate of the effect of romantic relationships on adolescent depression. In other words, adolescent romantic involvement may simultaneously increase depression—by violating social norms, undermining developmental outcomes, and increasing stress levels—and decrease depression—by enhancing positive emotional experiences, receiving emotional support from romantic partners, and fostering a greater sense of self-worth. The results of this study suggest that the potential negative consequences of romantic relationships outweigh their potential positive effects, leading to a net increase in depression. Nevertheless, the finding of a positive net effect highlights the necessity for targeted interventions, and thus holds significant practical relevance.

The bounding analysis further showed that estimates of the main effects of romantic relationships were not driven by omitted variable bias, which is contrary to Mendle et al.’s findings that romantic experiences did not have independent influence on adolescent depression and unobserved genetic and family environmental factors could explain the established correlations between the two (42). This result could be explained from the perspective of cultural context. Specifically, social norms regarding adolescent romance vary across cultures (6). In Western countries, although romantic involvement during early adolescence is considered non-normative behavior (43), it is not typically regarded as problematic. Moreover, Western families and schools tend to emphasize emotional expression and self-protection education rather than outright suppression of adolescent romantic behavior. In contrast, in China, adolescent romance—commonly referred to as “Zaolian” (precocious love)—is viewed as a deviant behavior throughout adolescence. It is often perceived as a distraction that undermines academic performance and adolescent development, and is frequently constrained and sanctioned by parents and teachers (6). Consequently, Chinese adolescents engaged in romantic relationships tend to experience greater psychological stress, resulting in a distinct, independent effect of romantic involvement on depression.

From a sociocultural development perspective, the differing attitudes toward adolescent romance between Chinese and Western societies can be viewed as a “cultural lag.” Specifically, although China has undergone rapid industrialization and economic growth, as a high power distance society, its traditional cultural values emphasizing control and obedience have yet to undergo commensurate transformation. This dynamic leads families and schools in China to intervene in adolescent romantic behavior primarily through control and punishment rather than through communication and supportive guidance. Such approaches not only fail to prevent romantic involvement but may also provoke severe psychological consequences, including self-harm and even suicide. Therefore, our findings underscore the importance of cultural context: the negative psychological impacts of adolescent romance are more likely to emerge in East Asian traditional societies that regard adolescent romance as problematic and adopt suppressive attitudes toward it (6). Correspondingly, as sociocultural transformations advance and support from schools and families improves, the adverse consequences of adolescent romantic involvement are expected to diminish gradually.

Additionally, collectivism, as another key feature of Chinese culture, suggests that the psychological impact of adolescent romantic relationships may also be shaped by social norms. Peers, as crucial reference groups during adolescence, exert significant regulatory influence on adolescents’ psychological well-being and behavior. Specifically, the prevalence of romantic involvement within peer networks (i.e., descriptive norms) not only provides adolescents with behavioral standards but also serves as an important alternative source for developing romantic competencies in the absence of adequate family and school support. This plays a key role in alleviating academic stress and interpersonal conflicts arising from romantic relationships. Meanwhile, peers’ attitudes toward adolescent romance (i.e., injunctive norms) determine the popularity and peer acceptance of adolescents engaged in romantic relationships, thereby influencing their exposure to social exclusion and stigma. Collectively, peer norms possess the potential to either amplify or buffer the psychological effects of adolescent romance. Building on this, the present study further examines the moderating role of peer norms in the association between adolescent romantic relationships and depression, aiming to provide empirical support for the prevention and intervention of adolescent mental health.

### 4.2 Moderating effects of peer norms

Overall, peer norms significantly moderated the link between romantic relationships and adolescent depression. Consistent with previous findings (9, 10), our findings indicated that descriptive norms serve as protective factors against depression in adolescents with romantic experiences. Social learning and social support could be potential mechanisms underlying the moderating effects of descriptive norms. Specifically, social learning theory posits that individuals adjust their behaviors and acquire new behavioral patterns by observing and imitating the actions of social referents (32). In the context of this study, adolescents embedded in peer networks where romantic involvement is more common may learn effective strategies for coping with the stress and challenges associated with romantic relationships (10). Social support theory further suggests that instrumental, informational, and emotional support from others can buffer the negative effects of stress, while enhancing self-esteem, psychological resilience, and self-regulation, thereby promoting mental health (44). In other words, peers with romantic experience may provide valuable support—such as advice and emotional comfort—to adolescents engaged in romantic relationships, thus mitigating the adverse impacts of romantic relationships on their psychosocial adjustment and mental health (45). It is crucial to note that the findings held not only for class norms but also for friend norms, contrary to van Zantvliet et al.’s conclusion that friend norms do not moderate the association between romantic involvement and internalizing problem behaviors (10). A possible explanation for this discrepancy is that van Zantvliet et al. limited their measurement of friend norms to friends within the class. But the friends who are more likely to provide social supports or to be imitated by adolescents may be outside class due to the streaming in secondary school, potentially leading to an underestimation of friend norms’ moderating role. In this paper, we estimated the moderating effects of friend norms more accurately by including friends outside the class, thereby contributing to the literature.

We further identified that injunctive norms played a significant moderating role between romantic relationships and adolescent depression. That is, the opposition of precocious love from peers intensifies the mental health costs of adolescents’ romantic involvement. Social influence could be potential mechanisms underlying the moderating effects of injunctive norms. Specifically, social influence theory posits that injunctive norms are closely tied to perceived social pressure, reflecting the likelihood of individuals being rewarded for engaging in normative behaviors or punished for engaging in deviant behaviors (46). Accordingly, when injunctive norms are characterized by disapproval of adolescent romantic relationships, adolescents who are romantically involved may face increased peer conflict and diminished social acceptance. These experiences can heighten perceived social stress and exacerbate the adverse effects of romantic involvement. In contrast, when peer groups do not oppose adolescent romance, romantic involvement may become a means for adolescents to demonstrate their attractiveness and elevate their popularity among peers, thereby facilitating psychosocial adjustment and mitigating the negative impacts of romantic relationships on well-being and mental health. Therefore, injunctive norms exert a positive moderating effect on the association between romantic relationships and adolescent depression, such that adolescents in romantic relationships not only suffer from the negative impacts of the relationship itself but also face social rejection for violating injunctive norms. This, in turn, leads to a heavier emotional burden and exacerbates the severity of depression.

Notably, Prior studies showed that adolescents were more sensitive to descriptive norms than injunctive norms (31, 32, 47), which may be attributed to their ability to circumvent injunctive norms without suffering sanctions through various strategies (48). While based on our findings, adolescents in a romantic relationship experienced more severe depression due to injunctive norms. The specific cultural environment could be a possible explanation. Adolescent romance is significantly impacted by distinct social norms in different cultural societies (21). In China, adolescent romance is widely seen as deviant behavior (49), leading to higher environmental pressures for Chinese adolescents in romantic relationships compared to those in Western cultures. Consequently, they tend to keep their relationships invisible to cope with this pressure and avoid seeking help from the outside world when experiencing emotional problems in their relationships, which further contributes to Chinese adolescents’ greater vulnerability to environmental pressures (33). Moreover, China’s highly competitive educational system is associated with significant academic stress, and poor academic performance is strongly correlated with elevated rates of adolescent depression (50). Engagement in romantic relationships not only directly impairs academic achievement but also indirectly undermines academic outcomes by reducing peer acceptance when peer networks hold opposing attitudes toward adolescent romance (51), thereby amplifying the adverse psychological effects of romantic relationships. Additionally, existing research indicates that family support can serve as an effective substitute for low peer acceptance, buffering the mental health risks associated with peer rejection (52). However, given the widespread perception in Chinese society that adolescent romance is problematic, parents typically adopt controlling and punitive approaches, leading to increased family conflicts for adolescents in romantic relationships (33). When such romantic involvement also violates peers’ injunctive norms, adolescents face a dual deficit in both family support and peer acceptance, further intensifying the negative psychological impacts of romantic relationships. The combined influence of these factors likely accounts for the stronger moderating effect of injunctive norms observed in China compared to Western cultures. Consequently, our findings expanded on the study of injunctive norms and teenage romantic relationships within the context of Chinese culture. However, it’s probable that our findings may not apply to adolescents in different cultural backgrounds. Future research should examine the moderating effects of injunctive norms based on other cultural contexts.

### 4.3 Practical implications for the prevention and intervention of adolescent depression

Our findings hold several implications for the prevention and intervention for adolescent depression. First, romantic relationships and depression often commence during adolescence. However, the impacts of romantic relationships on academic performance have received more attention than their effects on depression. In our study, we found that involvement in romantic relationships was associated with increased adolescent depression. This suggests that adolescent romance should be considered a critical target for the prevention and intervention of depression. Schools and families are advised to closely monitor the adolescent mental health, especially those in romantic relationships, and to help them develop social and love competence to mitigate the negative effects of such relationships. Specifically, schools can implement programs such as education on romantic relationships, emotional regulation training, and adolescent psychology seminars to enhance students’ social skills and support their healthy management of opposite-sex relationships. Simultaneously, a coordinated approach involving families, schools, and communities should be established to integrate parental education, school curricula, and community resources. This collaborative mechanism would enable close monitoring of adolescents’ mental health and provide guidance on addressing emotional challenges, ultimately contributing to a reduction in the incidence of adolescent depression.

Additionally, adolescence is featured by an expanded peer network and increased frequency of peer interactions (32, 43). Consequently, peer norms become an important source of influence on adolescents’ emotions and behaviors (31). Our findings showed that descriptive norms lessened the negative impact of romantic relationships on adolescent mental health, whereas injunctive norms exacerbated it. This offers implications for how to play the role of peer norms in the prevention and intervention of adolescent depression. Specifically, peer groups should be regarded as important models for disseminating coping strategies. Schools should develop school-based peer support programs integrated within the framework of school culture and mental health education. Such programs can employ group discussions and student clubs to teach skills such as active listening, emotional empathy, and conflict resolution, thereby enhancing adolescents’ psychological resilience and self-regulation. Additionally, teachers should establish positive classroom interaction mechanisms through activities like themed class meetings, “class support days,” and “worry suggestion boxes” to foster a supportive classroom environment. These initiatives strengthen peer support networks, enabling peers to provide necessary social support to students experiencing psychological difficulties, ultimately mitigating the adverse effects of romantic relationships and promoting adolescent mental health.

## 5 Limitations

Some limitations of our study need to be mentioned. First, this study employs cross-sectional data for empirical analysis, which limits our ability to estimate the long-term and dynamic effects of romantic relationships on adolescent depression. Future research should utilize subsequent waves of the CEPS data and apply methods such as cross-lagged panel models and latent growth curve models to examine the impact of romantic relationships on adolescent depression trajectories, as well as the moderating role of peer norms. Such approaches would overcome the current limitations and deepen our understanding of the psychological consequences of adolescent romantic relationships. Second, although this study addresses omitted variable bias through bounding approach, future research should adopt longitudinal designs or experimental methods to better establish causality. Specifically, subsequent studies could leverage future CEPS waves to construct panel data and employ fixed-effects models to control for time-invariant omitted variables. Additionally, quasi-experimental approaches using exogenous shocks, combined with instrumental variable and difference-in-differences methods, could provide stronger causal evidence. Furthermore, experimental designs such as vignette studies or priming interventions could be utilized to enhance causal inference. Third, due to the sensitive nature of adolescent romantic relationships in the Chinese context and the need to protect adolescents’ privacy, the 2014–2015 CEPS did not include detailed questions related to romantic relationships. Consequently, this study only examined the impact of whether adolescents were engaged in a romantic relationship on depression, without empirically exploring the potential effects of factors such as relationship duration, emotional experiences, emotional support provided by partners, and key events during the relationship (e.g., the experience of sudden heartbreak and the dynamics of conflict). Future research should, with informed consent from both students and their parents, collect such data through questionnaires or in-depth interviews, enabling the use of quantitative or qualitative methods to explore the effects of these factors on adolescent depression, as well as the moderating role of peer norms. Fourth, limited by data availability, this study examined the mechanisms underlying the moderating effects of peer norms only from a theoretical perspective, without empirical testing. Future research should employ a combination of quantitative and qualitative methods to further differentiate among various types of peer norms, deepen the understanding of their respective mechanisms, and distinguish the relative importance of potential pathways such as social learning, social support, and social influence. Such efforts will enhance the precision and effectiveness of prevention and intervention strategies targeting adolescent depression. Finally, we measured injunctive norms with adolescents’ perceived peer attitudes, which may lead to mismeasurement of the moderating effects of injunctive norms. For instance, adolescents might exhibit social desirability bias when reporting perceived injunctive norms in order to maintain their self-image and avoid negative evaluation, thereby obscuring their true beliefs about peer attitudes. This bias is likely to be amplified when addressing sensitive topics such as romantic relationships, potentially compromising the accuracy of the estimated moderating effects of injunctive norms. Future research should consider employing methods such as the item count technique or using peers’ actual attitudes to measure injunctive norms, thereby reducing social desirability bias, minimizing measurement error, and enhancing the validity of the findings.

## 6 Conclusions

In summary, our investigation supports that romantic relationships significantly positively predict adolescent depression in China. Our results also suggest that peer norms, including both descriptive norms and injunctive norms, have significant moderating effects on the relations between romantic relationships and adolescent depression. In other words, the negative effects of romantic relationships are attenuated when romantic involvement is more common in peer networks, and intensified when peers are opposed to adolescence romance.

## Data Availability

Publicly available datasets were analyzed in this study. This data can be found here: http://ceps.ruc.edu.cn/.
